# Supplementary material for: Peroxiredoxin 6 mediates Gαi protein-coupled receptor inactivation by cJun kinase
Source: Nat Commun. 2017 Sep 29;8:743. doi: 10.1038/s41467-017-00791-2 (PMC5622097; doi:10.1038/s41467-017-00791-2)
Supplement: Supplementary file 1 — Supplementary Information [file 41467_2017_791_MOESM1_ESM.pdf]

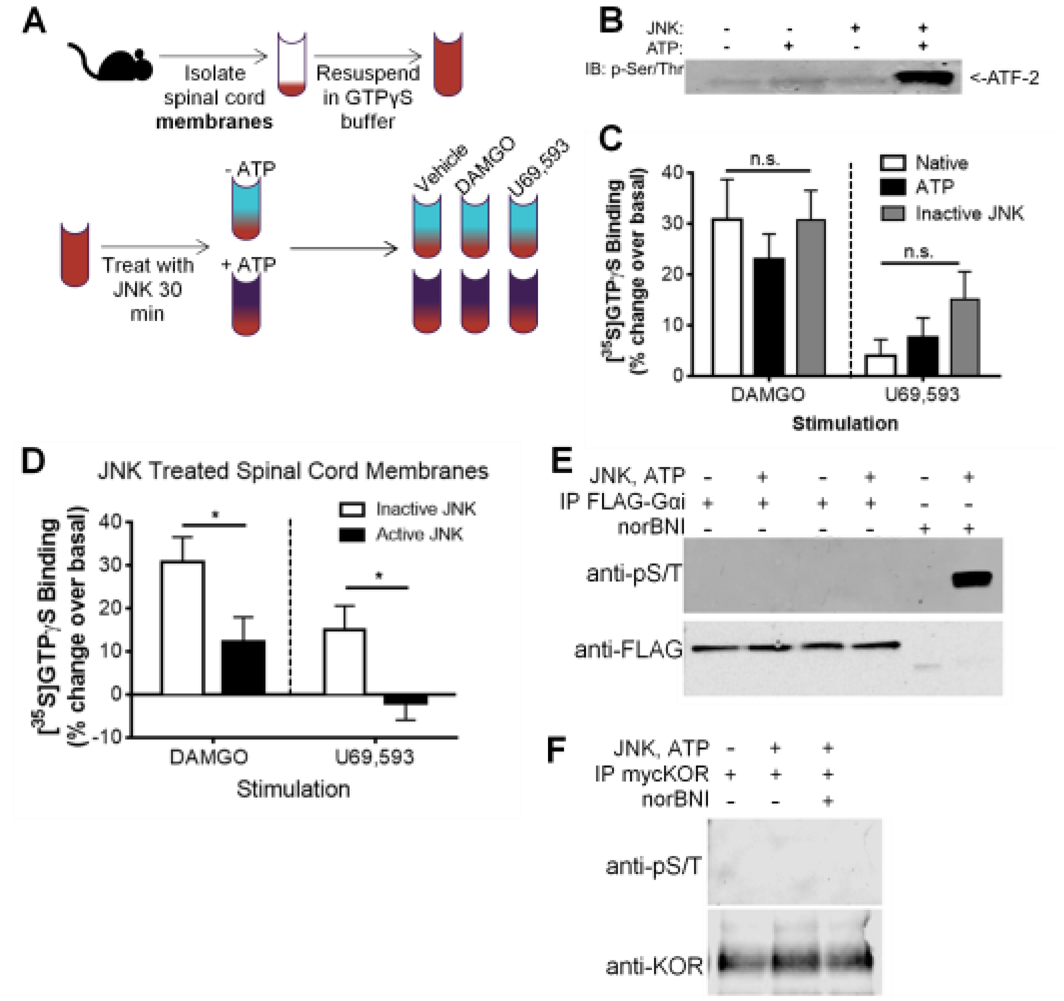

Supplementary Figure 1. *In vitro* JNK inhibits mu and kappa opioid receptor stimulated GTPγS binding. Relates to Figure 1. (A) Schematic of in vitro kinase $[^{35}S]$GTPγS binding assay. (B) *In vitro* JNK-stimulated GST-ATF-2 phosphorylation was observed after 30 min *in vitro* JNK stimulation only when incubated with both ATP and JNK1 enzyme. (C) DAMGO and U69,593 stimulated $[^{35}S]$GTPγS binding in mouse spinal cord membranes were not significantly different between native membranes and membranes treated with JNK in the absence of ATP or membranes treated with ATP in the absence of JNK (Student's t-test without corrections, n=11-20). (D) In contrast, DAMGO and U69,593 stimulated $[^{35}S]$GTPγS binding in spinal cord membranes were significantly reduced following treatment with *in vitro* JNK1 and ATP, but not *in vitro* JNK alone. (Student's t-test, $p<0.05$, n=11). (E) *In vitro* JNK treatment did not stimulate phosphorylation of FLAG-Gαi3 under conditions that stimulated ATF2 phosphorylation and was also not observed when the kinase reaction was supplemented with 25nM GDP or GTP. (F) *In vitro* JNK treatment did not increase phosphorylation of mycKOR (in the absence or presence of 10 µM norBNI) under conditions that increased ATF2 phosphorylation. Error bars represent mean±SEM.

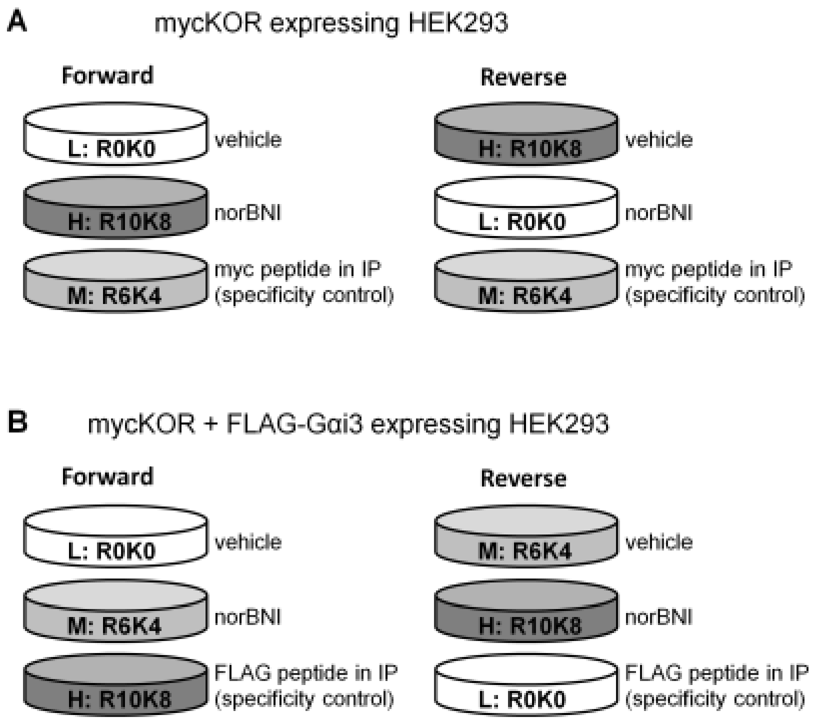

Supplementary Figure 2. SILAC experimental design. Relates to Figure 1a, Figure 2a, Supplementary Data 1, and Supplementary Data 2. (A) Schematic of stable isotope labeling and drug treatment protocol for the SILAC experiments in Figure 1A and Supplementary Data 1. Isotope labeling is indicated as L:R0K0 ("light", arg0/lys0), M:R6K4 ("medium", arg6/lys4), H:R10K8 ("heavy", arg10/lys8). MycKOR expressing HEK293 cells were grown in stable isotope labeled media, treated with vehicle or norBNI, and prepared as described in the methods. In the 'forward' reaction, heavy amino acid labeled cells were treated with norBNI (10 µM, 5.5 hr) and 'light' were treated with vehicle before membrane proteins were detergent solubilized and mycKOR was isolated by immunoprecipitation with anti-myc agarose. In the 'reverse' reaction, the 'light' labeled HEK293 cells were treated with vehicle and the 'heavy' labeled cells were treated with 10 µM norBNI. To identify nonspecific binding, 'medium' labeled cells were treated with norBNI prior to immunoprecipitation in the presence of excess myc protein. (B) Schematic of isotope labeling and drug treatment protocol for the SILAC experiments shown in Figure 2A and Supplementary Data 2. MycKOR and FLAG-GαI3 expressing HEK293 cells were grown in isotope labeled media, treated with vehicle or norBNI, and prepared as described in the methods. In the 'forward' reaction, 'medium' labeled cells were treated with norBNI (10 µM, 5.5 hr) and 'light' were treated with vehicle before membrane proteins were detergent solubilized and FLAG-Gαi3 was immunoprecipitated with anti-FLAG agarose. In the 'reverse' reaction, the 'medium' labeled HEK cells were treated with vehicle and the 'heavy' labeled cells were treated with norBNI. To assess nonspecific binding, 'heavy' (forward reaction) and 'light' (reverse reaction) labeled cells were treated with norBNI prior to immunoprecipitation in the presence of excess FLAG protein.

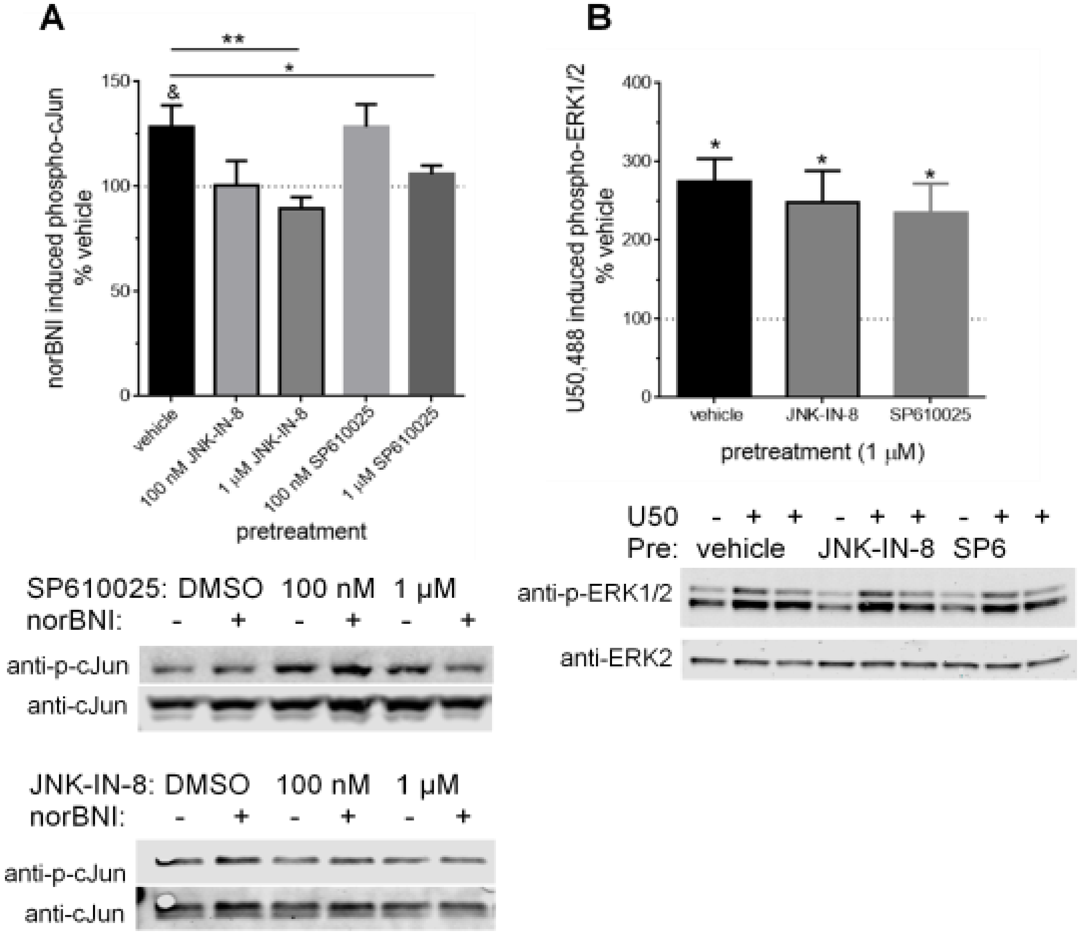

Supplementary Figure 3. Relates to Figure 1, 2, and 3. HEK293 cells expressing mycKOR were pretreated with vehicle, JNK-IN-8, or SP610025 30 min prior to 90 min treatment with 10 µM norBNI (A) or 5 min treatment with 1 µM U50,488 (B, C). (A) 1 µM JNK-IN-8 and SP610026 significantly blocked the norBNI-induced increase in phospho-cJun IR (& $p<0.05$, one-sample t-test compared with match vehicle control); *($p<0.05$), ** ($p<0.01$); Student's t-test); *n*=5-11. (B) 1 µM JNK-IN-8 and SP610026 has no effect on U50,488 stimulated phospho-ERK1/2 IR (&, one-sample t-test; n.s., Student's t-test; *n*=6-7). Error bars represent mean±SEM.

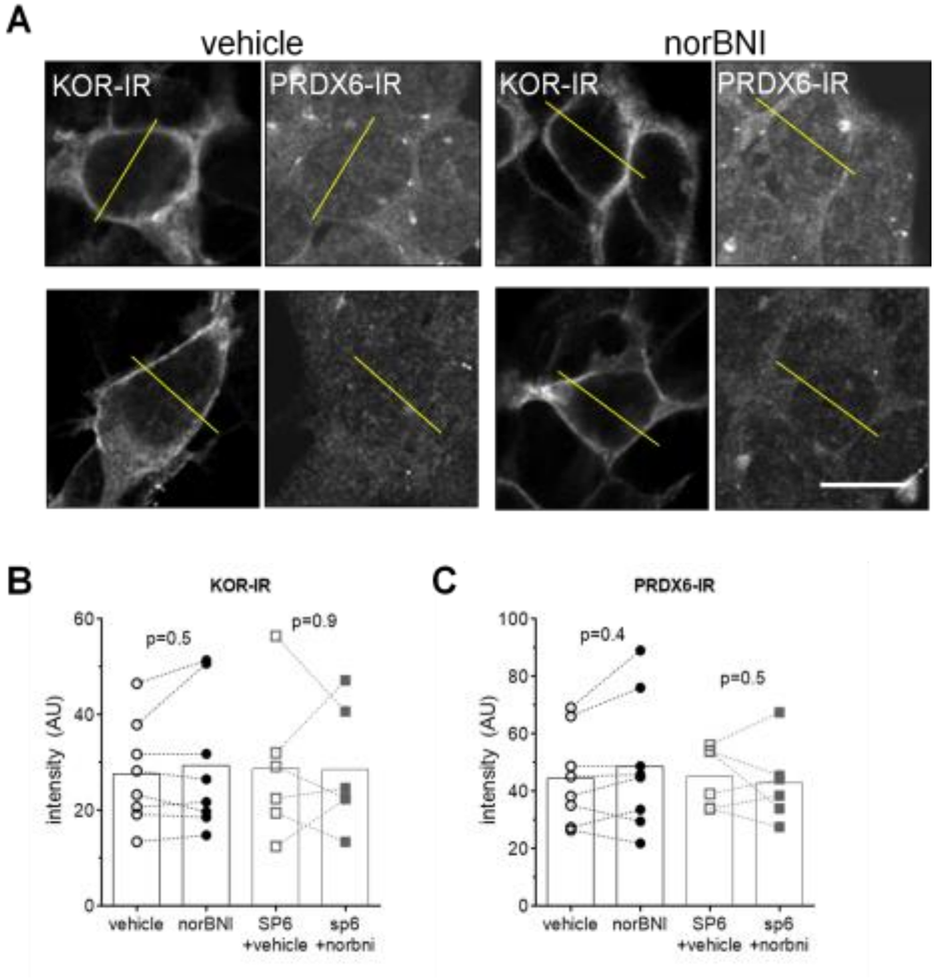

Supplementary Figure 4. Relates to Figure 2F. (A) MycKOR expressing cells were immunostained for KOR and PRDX6. Colocalization was quantified by correlation between intensity of KOR and PRDX6 immunoreactivity across 7-10 cells for each replicate; representative cells are shown; scale bar represents 20 µm. Quantification is presented in Figure 2. (B,C) The average intensity of KOR (B) and PRDX6 (C) immunoreactivity was calculated from the line plot analysis data in Figure 2F. No significant changes in immunofluorescence was observed (Student's paired t-test, *n*=6-8). Error bars represent mean±SEM.

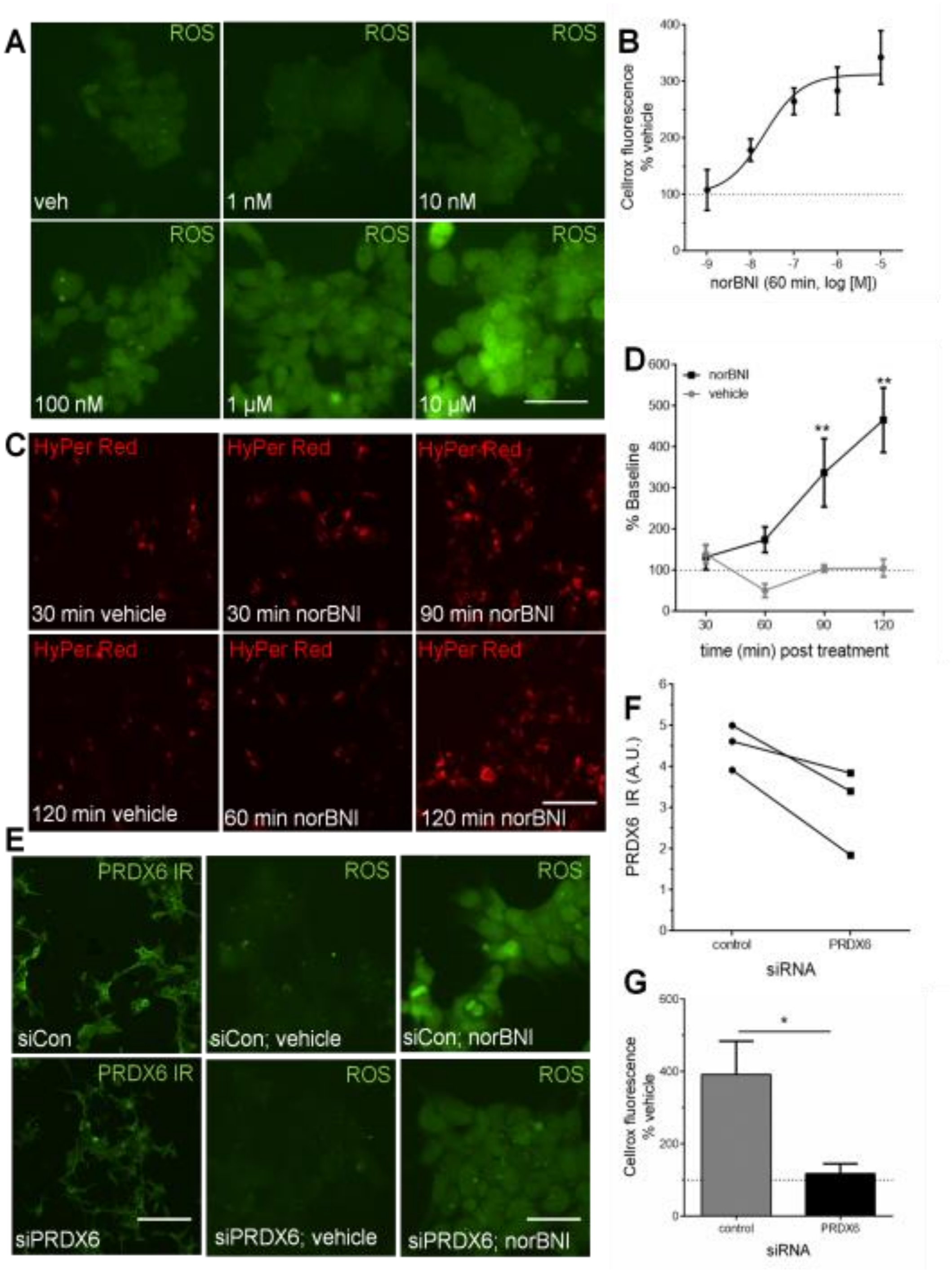

Supplementary Figure 5. Relates to Figure 3. (A, B) MycKOR expressing HEK293 were treated for 1 hr with 1 nM - 10 µM norBNI and imaged for ROS using CellROX Green; scale bar represents 12.5 µm. NorBNI induces a concentration dependent increase in CellROX Green fluorescence, with an EC50 of 21 nM (95% confidence interval 5.0-86 nM; three parameter least-squares non-linear regression; *n*=3). (C, D) MycKOR HEK293 cells transiently expressing the ROS sensor HyPer Red 30 hr prior to 30-120 min treatment with 10 µM norBNI or vehicle;

scale bar represents 50 µm. Data are presented as % baseline, calculated as the average fluorescence across all vehicle time points within a replicate. A significant increase in HyPerRed fluorescence is observed by 90 min (two-way ANOVA; significant effect of time ($p<0.01$), norBNI ($p<0.0001$), and interaction; ** ($p<0.01$) Holm-Sidak post-hoc; *n*=3). (E-G). MycKOR HEK293 cells were transfected with siRNA against PRDX6 or control siRNA 36-40 hr prior to treatment with 10 µM norBNI for 60 min, and imaged for ROS using CellROX Green; scale bar represents 50 µm (PRDX6 IR) or 12.5 µm. (E, F) PRDX6 siRNA reduced PRDX6 IR by 34% ($p=0.06$, paired t-test). PRDX6 siRNA significantly inhibited the norBNI-stimulated increase in CellROX Green fluorescence (* ($p<0.05$), student's t-test; *n*=4-5). No significant effect of PRDX6 siRNA on basal fluorescence was observed (PRDX6 siRNA 123±31% of control siRNA). Error bars represent mean±SEM.

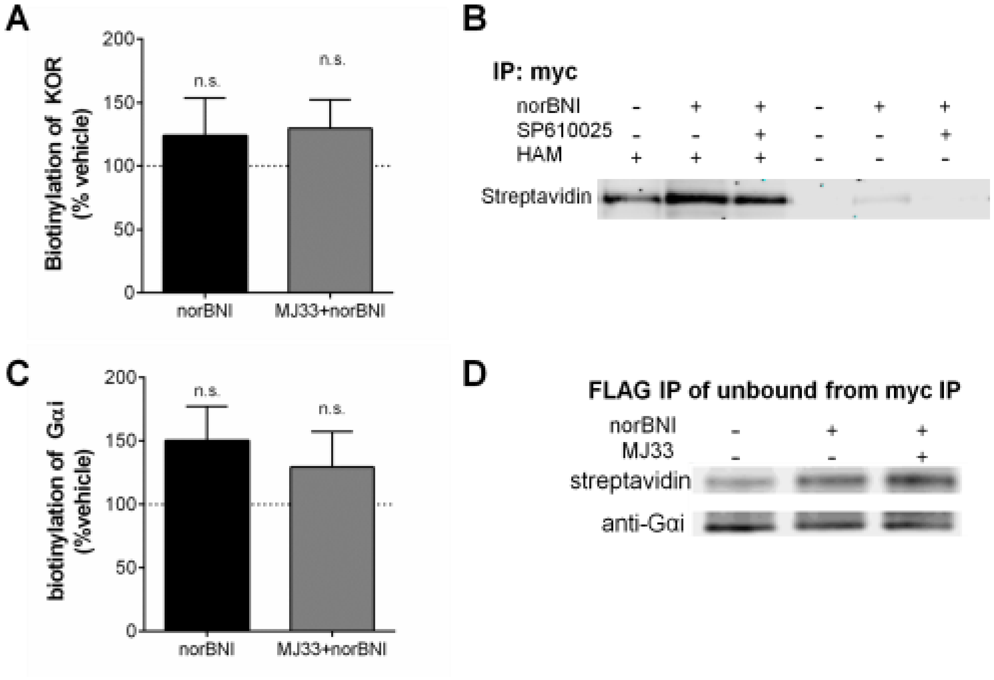

Supplementary Figure 6. Relates to Figure 3E-G. HEK293 cells stably co-expressing mycKOR and FLAG-Gαi3 were pretreated with vehicle, SP610025, or MJ33 and treated 5.5 hr with norBNI prior to harvest. Cell membranes were extracted in the presence of N-ethylmaleimide (50 mM) to covalently bind unmodified cysteines, and immunoprecipitated with anti-myc to isolate mycKOR and KOR-associated Gαi3. Proteins not immunoprecipitated with anti-myc were incubated with anti-FLAG agarose to isolate the population of Gαi3 not bound to KOR. Myc and FLAG immunoprecipitates were treated with hydroxylamine to cleave palmitic acids and then incubated with BMCC-biotin to biotinylate previously palmitoylated cysteines. Palmitoylation was measured by probing with streptavidin and normalizing to anti-Gαi immunoreactivity. (A) NorBNI did not change palmitoylation of mycKOR (one sample t-test, p=0.47, *n*=5). (B) Representative immunoblots for A; minimal biotinylation is detected in absence of hydroxylamine treatment. (C) NorBNI did not change palmitoylation of Gαi3 which were not co-immunoprecipitated with mycKOR (one sample t-test, p=0.12, *n*=6). (D) Representative immunoblots for C. Error bars represent mean±SEM.

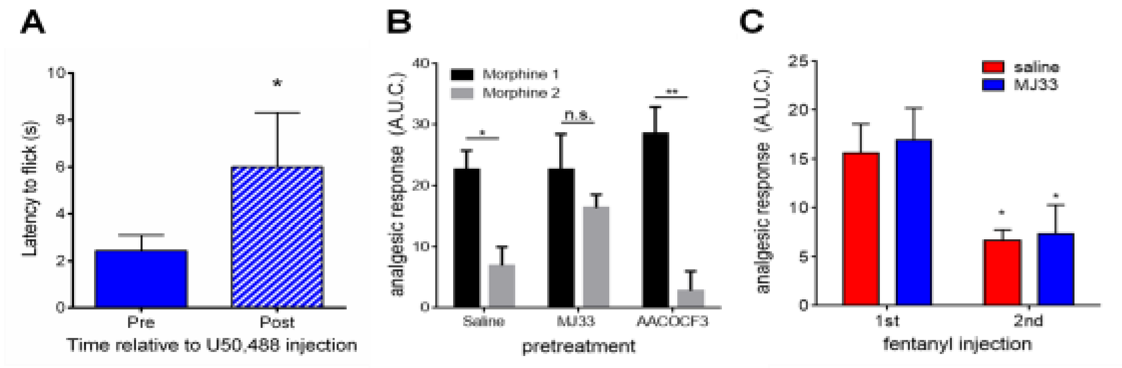

Supplementary Figure 7. Relates to Figure 4. (A) Mice were injected with MJ33 (1.25 mg kg^{-1}, i.p.) 1 hr prior to injection with U50,488 (10 mg kg^{-1}, i.p.). MJ33 did not change the U50,488-stimulated increase in latency (ratio-paired student's t-test, $p<0.05$). Mice were injected with saline or MJ33 or AACOCF3 (10 mg kg^{-1}, i.p.) 4 hr prior to the initial injection of morphine (10 mg kg^{-1}, i.p.) (B, C) Mice were injected with saline or MJ33 4 hr prior to the initial injection of morphine (10 mg kg^{-1}, i.p.) or fentanyl (0.3 mg kg^{-1}, i.p.). Tail withdrawal latency was measured prior to and at 30 min intervals following morphine or fentanyl. To detect acute analgesic tolerance, after latency returned to baseline a second agonist injection was administered. Area-under the curve for the data in Figure 4C,D was calculated. (B) A reduction in analgesic response to the 2nd injection of morphine was observed following saline pretreatment and AACOCF3 pretreatment, but not MJ33 pretreatment (paired two-way ANOVA; significant effect of injection; * ($p<0.05$), ** ($p<0.01$) Holm-Sidak post-hoc; *n*=6-8). (C). The analgesic response to the second injection of fentanyl was significantly reduced following both saline and MJ33 pretreatment (paired two-way ANOVA; significant effect of time ($p<0.01$); * ($p<0.05$) Holm-Sidak post-hoc; *n*=6-7). Error bars represent mean±SEM.

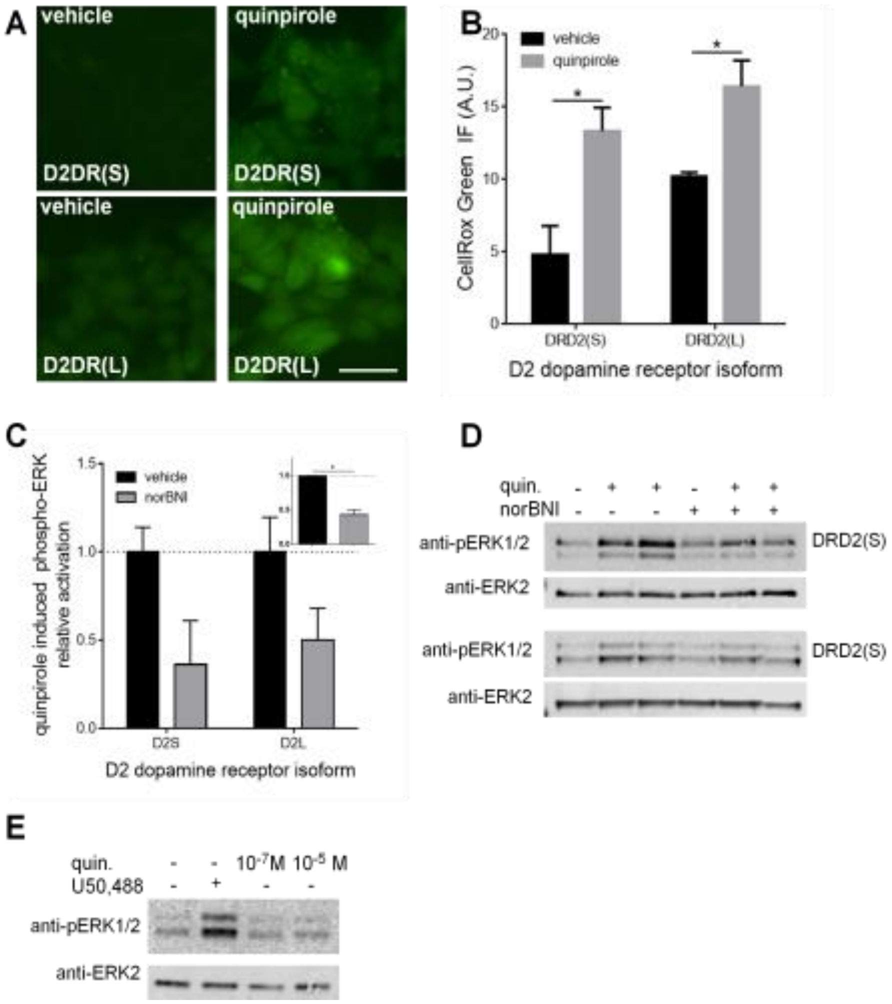

Supplementary Figure 8. Relates to Figures 5, 6. (A) HEK293 cells transiently expressing with HA-tagged D2DR(S) or DRD2(L) were treated 60 min with 100 nM quinpirole and imaged for ROS using CellROX Green; scale bar represents 10 µm. (B) Quantification of data in A. Quinpirole increased CellROX Green fluorescence in cells expressing either short and long isoforms of the D2 dopamine receptor (two way ANOVA; significant effect of isoform ($p<0.05$) and drug ($p<0.01$); *($p<0.05$) Holm-Sidak post-hoc; $n = 3$). (C,D) HEK293 cells stably expressing mycKOR and transiently expressing HA-DRD2(S) or HA-D2DR(L) were treated 3-5 hr with vehicle or norBNI prior to treatment for 5 min with 100 nM quinpirole. Cell lysates were analyzed for phospho-ERK1/2 IR. NorBNI treatment reduced quinpirole stimulated ERK1/2 phosphorylation in cells expressing either receptor isoform (two-way ANOVA; significant effect of norBNI ($p<0.05$); $n = 4$-6). (E) HEK293 cells stably expressing mycKOR were treated 5 min with 100 nM or 10 µM quinpirole or 1 µM U50,488. Cell lysates were analyzed for phospho-ERK1/2 IR. U50,488, but not quinpirole, stimulated ERK1/2 phosphorylation. (Phospho-ERK1/2 IR averaged 85±5% and 123±47% of vehicle following 100 nM and 10µM quinpirole, respectively; n=3.) Error bars represent mean±SEM.

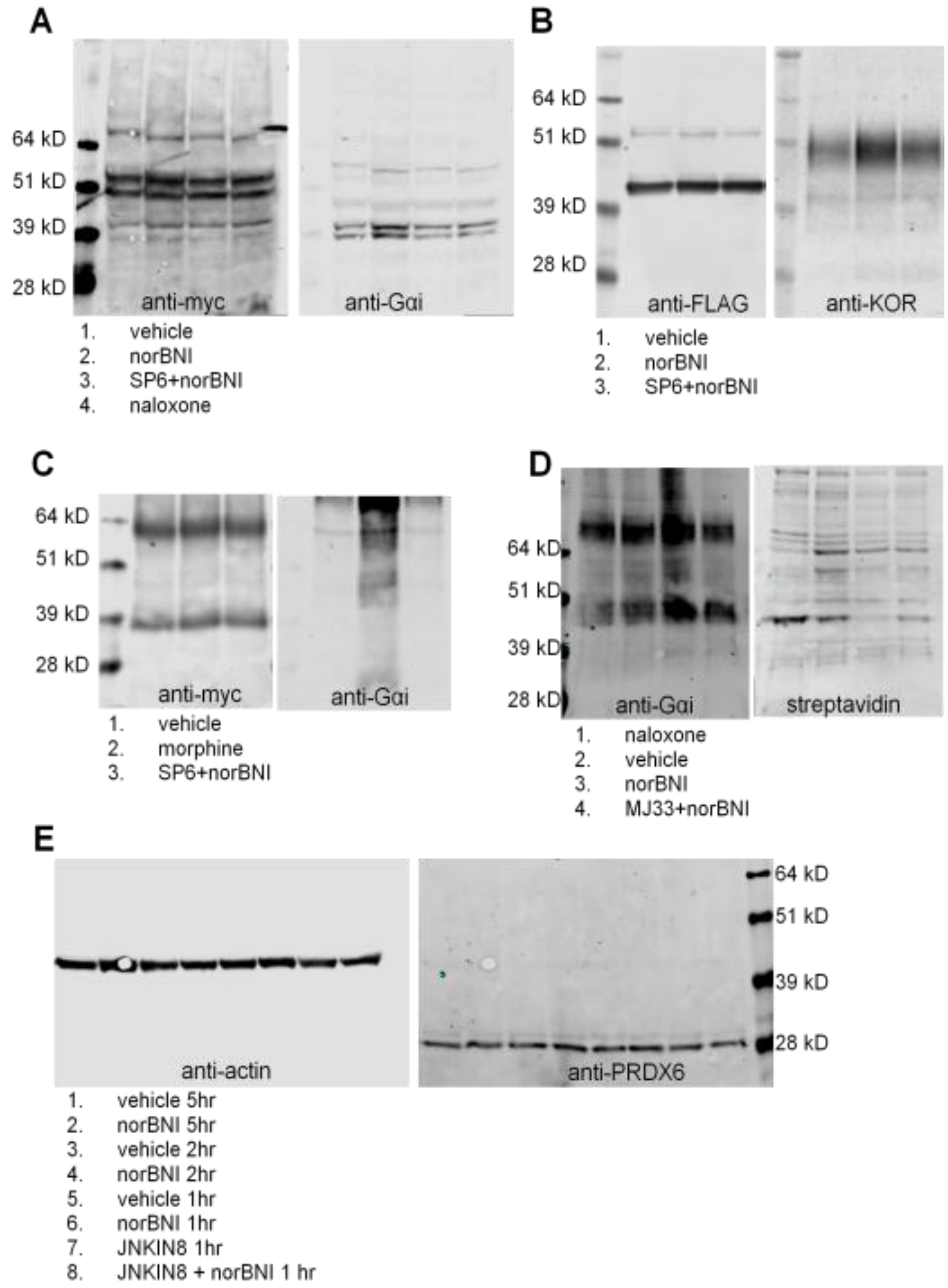

Supplementary Figure 9. Relates to Figures 1,2, and 3. (A) Western blot from Figure 1B. (B) Western blot from Figure 1B. (C) Western blot from Figure 1C. (D) Western blot from Figure 3G. (E) Western blot from Figure 2H.

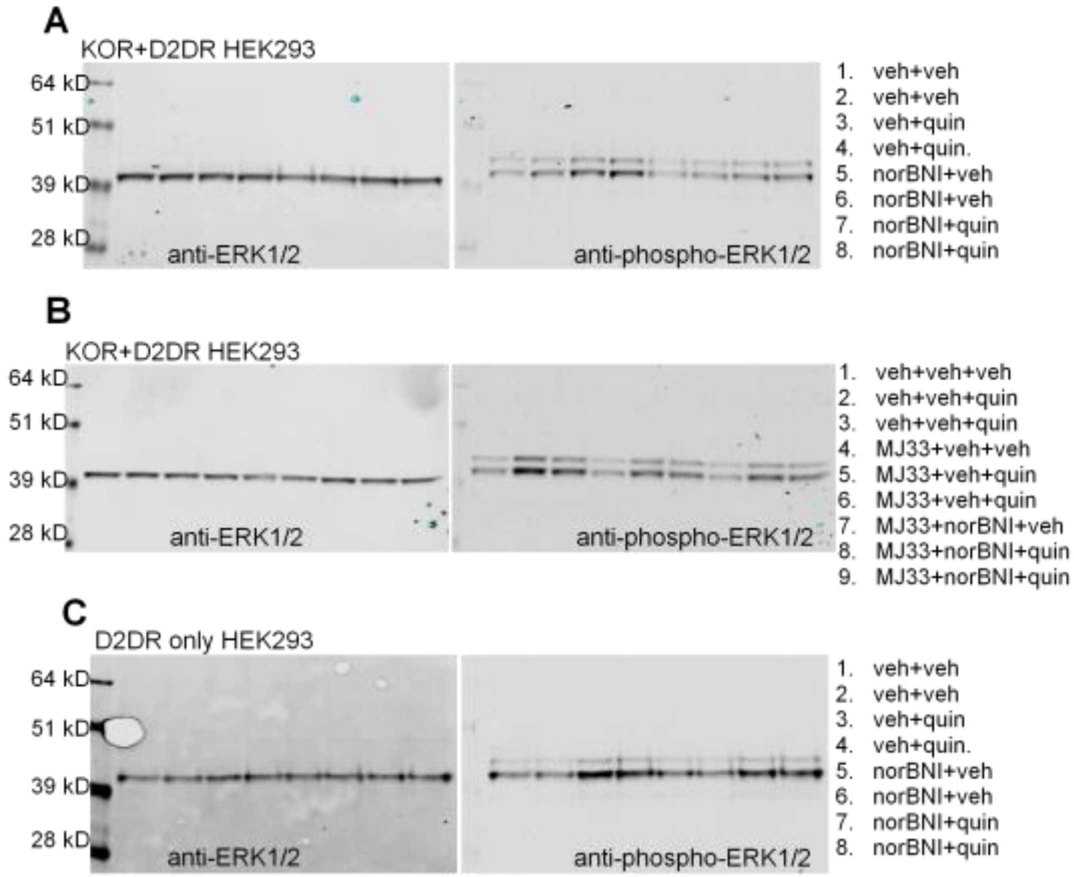

Supplementary Figure 10. Relates to Figure 6B. Western blots from Figure 6B depicting norBNI inhibition quinpirole stimulated ERK1/2 phosphorylation in the absence of MJ33 (A), presence of MJ33 (B), or the absence of KOR (C).
