# Supplementary material for: Peroxiredoxin 6 mediates Gαi protein-coupled receptor inactivation by cJun kinase
Source: Nat Commun. 2017 Sep 29;8:743. doi: 10.1038/s41467-017-00791-2 (PMC5622097; doi:10.1038/s41467-017-00791-2)
Supplement: Supplementary file 2 — Description of Additional Supplementary Files [file 41467_2017_791_MOESM2_ESM.pdf]

Description of Additional Supplementary Files

File Name: Supplementary Data 1

Description: NorBNI induced changes in mycKOR-interacting proteins. Relates to Figure 1. MaxQuant software analysis results comparing KOR-interacting proteins are summarized. All proteins listed were enriched relative to competition myc peptide control, and are listed in order of decreasing enrichment upon norBNI treatment, averaged between the forward and reverse replicate. Gene names with Genecard link and protein name are provided along with counts, average ratio and FDR-based significance B score for the identified peptides for each protein. The total spectral intensity for each identified protein is listed for vehicle and norBNI treated conditions. * Protein significantly changed by norBNI as compared to vehicle. (log2 of ratio >0.5 and Significance B

File Name: Supplementary Data 2

Description: NorBNI induced changes in FLAG-Gαi3-interacting proteins. Relates to Figure 2. MaxQuant software analysis results comparing Gαi-interacting proteins are listed. All proteins listed were significantly enriched relative to competition FLAG peptide control, and are listed in order of decreasing enrichment upon norBNI treatment, averaged between the forward and reverse replicate. Gene names with Genecard link and protein name are provided along with counts, average ratio and FDR-based significance B score for the identified peptides for each protein. The total spectral intensities for each identified protein are listed for vehicle and norBNI treated conditions. A total of 76 proteins were identified as FLAG-Gαi3 interactors; 54 were identified in under both basal and norBNI treated conditions, while 18 were identified following vehicle treatment only, and 4 were identified in norBNI treated conditions only
